# New information on the Wukongopteridae (Pterosauria) revealed by a new specimen from the Jurassic of China

**DOI:** 10.7717/peerj.2177

**Published:** 2016-07-07

**Authors:** Xin Cheng, Shunxing Jiang, Xiaolin Wang, Alexander W.A. Kellner

**Affiliations:** 1 Key Laboratory of Vertebrate Evolution and Human Origins of Chinese Academy of Sciences, Institute of Vertebrate Paleontology and Paleoanthropology, Beijing, China; 2 University of Chinese Academy of Sciences, Beijing, China; 3 Department of Geology and Paleontology, National Museum/UFRJ, Rio de Janeiro, Brazil

**Keywords:** Wukongopteridae, Pterosauria, Yanliao Biota, China, Late Jurassic

## Abstract

The Wukongopteridae is an important pterosaur group discovered from Yanliao Biota, because it combines character states seen in non-pterodactyloid and pterodactyloid pterosaurs. So far, the Wukongopteridae contains three genera: *Wukongopterus*, *Darwinopterus* and *Kunpengopterus*; representing five species. Here we report on a new specimen, IVPP V 17959, that can be undoubtedly referred to the Wukongopteridae based on the presence of a confluent nasoantorbital fenestra, elongated cervical vertebrae (convergent with Pterodactyloidea) and a long tail enclosed by rod-like bony extensions of the zygapophyses. Traits distinguishing this new specimen from other wukongopterid pterosaurs include a premaxilla with a low ossified anterodorsal crest, a nasal bearing the most elongated process known in the Wukongopteridae, and a lacrimal that has a foramen in its middle portion. The new kind of premaxillary crest preserved in IVPP V 17959 suggests that the presence or absence of a premaxillary crest might be an interspecific feature within the Wukongopteridae. A phylogenetic analysis including all wukongopterid pterosaurs recovers IVPP V 17959 in a polytomy with *Wukongopterus* and the species of *Darwinopterus*, having* Kunpengopterus* in a more basal position. The postcranial skeleton of IVPP V 17959 has ontogenetically mature characteristics including a completely fused scapula and coracoid, fused proximal and distal carpal series, and an ossified extensor tendon process of the first wing phalanx, allowing its classification as ontogenetic stage five. Furthermore, the atlas and axis are separated in IVPP V 17959, which indicates that these two bones probably are not fused in skeletally mature wukongopterid individuals.

## Introduction

Over the last ten years, new discoveries of pterosaurs from China have been important for understanding the taxonomy, morphology, and life history (e.g., reproduction) of the clade (e.g., Andres, Clark & Xu, 2010; Andres, Clark & Xu, 2014; Wang et al., 2014b; Wang et al., 2015). Many new taxa have been discovered, mostly coming from northeast China, including the deposits of the Early Cretaceous Jehol Biota and the Late Jurassic Yanliao Biota (e.g., Cheng et al., 2012; Wang et al., 2012; Wang et al., 2014c; Cheng et al., 2015; Rodrigues et al., 2015).

The Wukongopteridae is a noteworthy group of the Linglongta Pterosaur Fauna, which is a part of Yanliao Biota (Zhou & Wang, 2010; Cheng, 2013; Sullivan et al., 2014). This non-pterodactyloid clade combines character states evident in both non-pterodactyloid and pterodactyloid pterosaurs (Wang et al., 2009; Lü et al., 2010; Wang et al., 2010). Currently, three genera and five species of wukongopterid pterosaurs have been described (Wang et al., 2009; Lü et al., 2010; Wang et al., 2010; Lü et al., 2011b), including a gravid female with two eggs (Lü et al., 2011a; Wang et al., 2015). According to Lü et al. (2011a), crested wukongopterids were male and crestless ones were female individuals, but this idea was later challenged by Wang et al. (2015).

Here we report on a new wukongopterid specimen (IVPP V 17959) from Linglongta, which provides new information on the morphology of the premaxillary crest, that along with other observations suggest that this structure might be interspecific rather than sexually dimorphic at least within this clade of non-pterodactyloid pterosaurs.

## Materials and Methods

**Figure 1:**
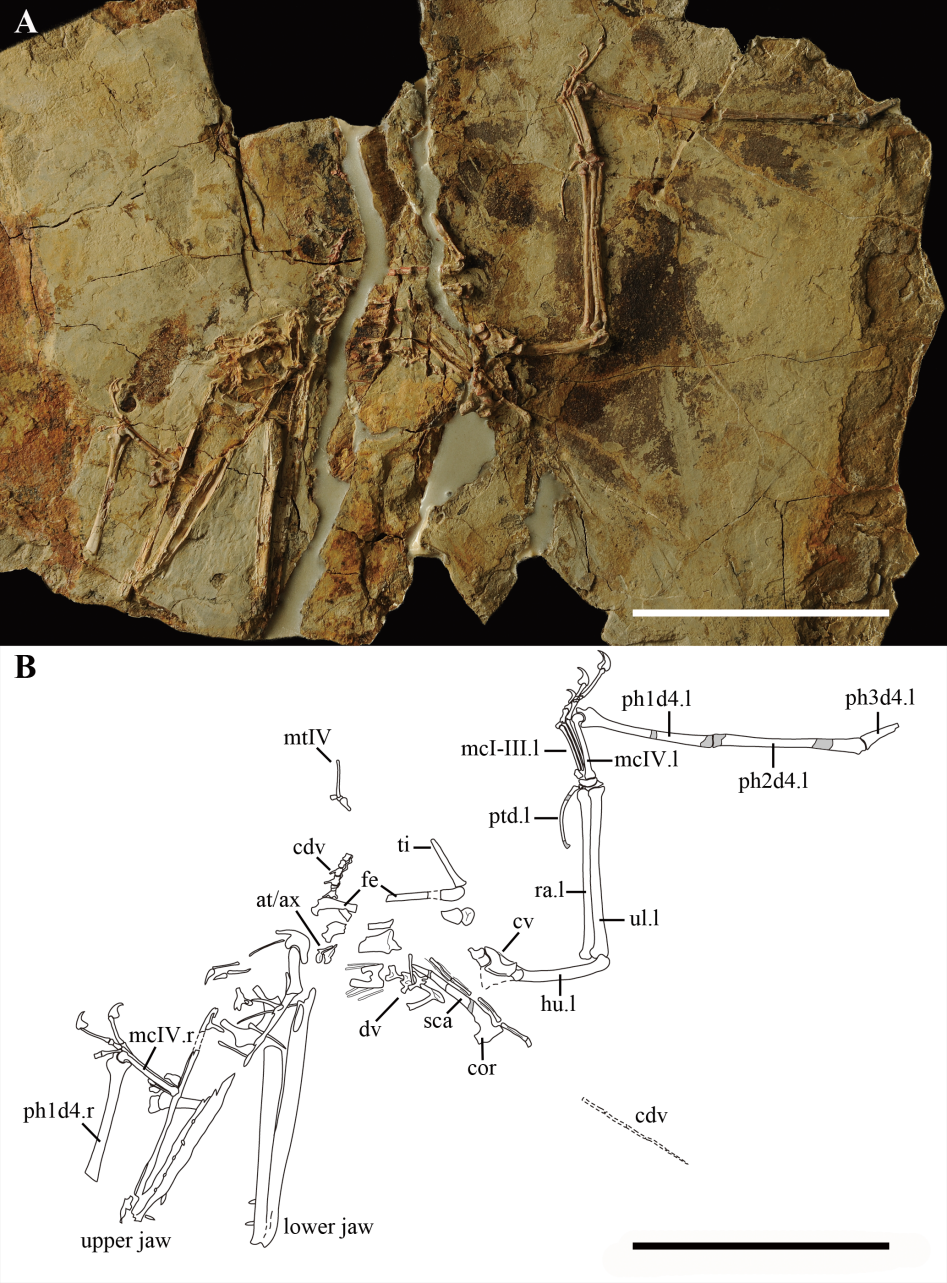
IVPP V 17959 that preserved in a slab of grey-white shale. (A) Photo and (B) Line drawing of the complete skeleton with the impression part in grey. Scale bar: 100 mm. Abbreviation: at, atlas; ax, axis; cdv, caudal vertebra; cor, coracoid; cv, cervical vertebra; dv, dorsal vertebra; fe, femur; hu, humerus; l, left; mcI-IV, metacarpal 1–4; mtIV, metatarsal 4; ph1-3d4, first to third phalanx of digit 4; ptd, pteroid; r, right; ra, radius; sca, scapula; ti, tibia; ul, ulna.

IVPP V 17959 consists of an incomplete skeleton of a skeletally mature individual with the skull preserved in left view in a single slab (Fig. 1) that was discovered from the Yanliao deposits near Daxishan (Jianchang, Liaoning Province, China). The specimen was collected by a local resident and is presently housed in the Institute of Vertebrate Paleontology and Paleoanthropology, Chinese Academy of Sciences (IVPP/CAS). IVPP V 17959 came to IVPP divided into several parts that were glued together using epoxy glue and was then prepared mechanically by needle and pneumatic micro tools under a microscope.

Our phylogenetic analysis was done with TNT (Tree Analysis Using New Technology) for Microsoft Windows (Goloboff, Farris & Nixon, 2008) using the Traditional Search, with the Maxtree setting as 10,000. All characters were given equal weight and treated as unordered. This analysis was mainly based on the phylogenetic analysis published by Wang et al. (2012), including some taxa used in previous analysis (Kellner, 2003; Wang et al., 2009; Lü et al., 2010; Supplemental Information 1). Furthermore, some non-pterodactyloid taxa and all wukongopterid pterosaurs were added with the exception of the purported European wukongopterid (Martill & Etches, 2013), which lacks postcranial material. The matrix contained 106 characters and 43 taxa (including three outgroups as: *Ornithosuchus longidens*, *Herrerasaurus ischigualastensis* and *Scleromochlus taylori*).

## Results

### Systematic paleontology

**Table:** 

PTEROSAURIA Kaup, 1834
WUKONGOPTERIDAE Wang et al. (2009)

***Specimen***

**Figure 2:**
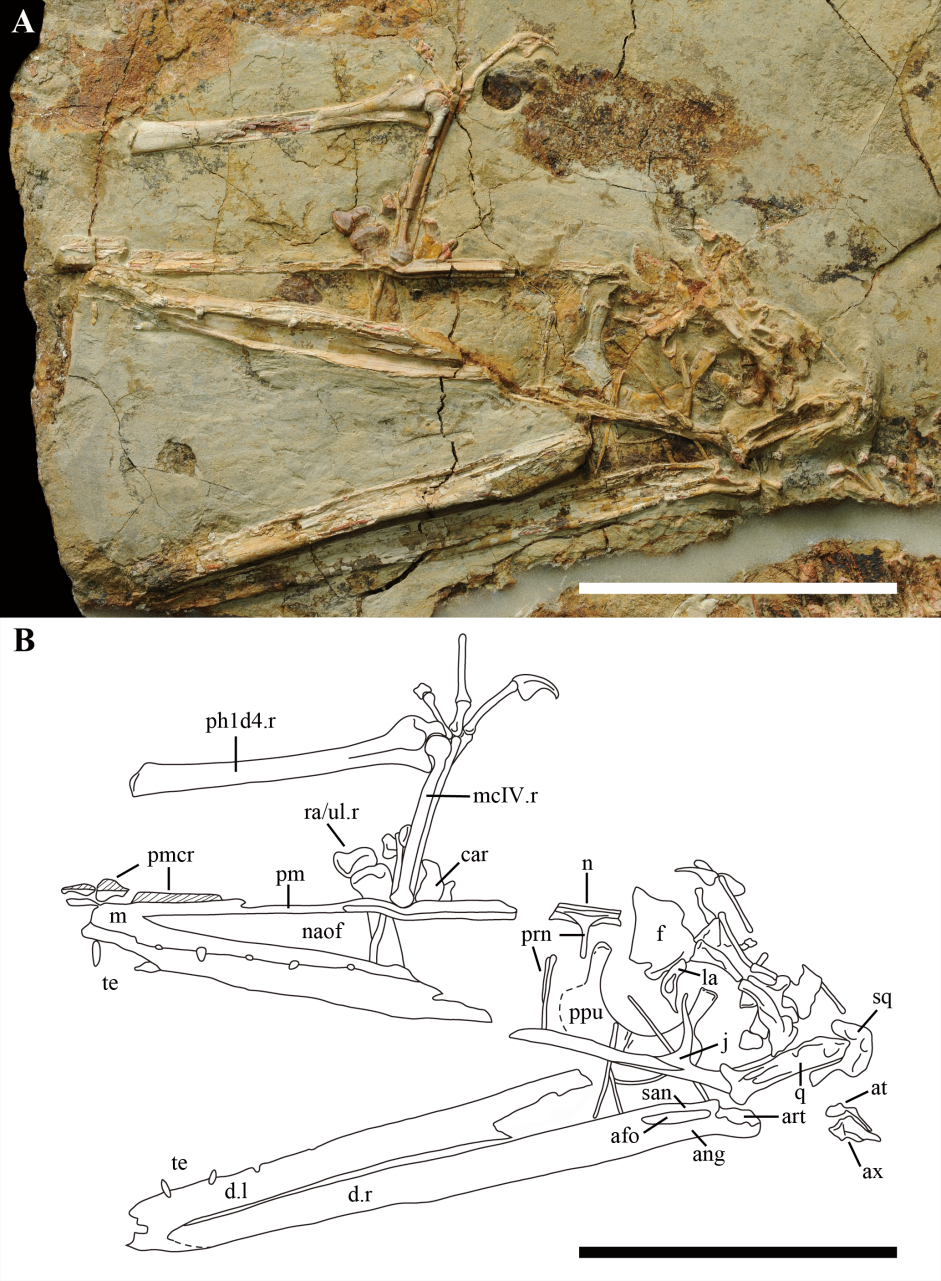
Details of the skull in IVPP V 17959. (A) Close up of the skull. (B) Line drawing of the skull. Scale bar: 50 mm. Abbreviation: afo, adductor fossa; ang, angular; art, articular; at, atlas; ax, axis; car, carpal; d, dentary; f, frontal; j, jugal; l, left; la, lacrimal; m, maxilla; mcIV, metacarpal 4; n, nasal; naof, nasoantorbital; ph1d4, first phalanx of digit 4; pm, premaxilla; pmcr, premaxilla crest; ppu, prepubis; prn, process of nasal; q, quadrate; ra, radius; r, right; san, surangular; sq, squamosal; te, teeth; ul, ulna.

**Figure 3:**
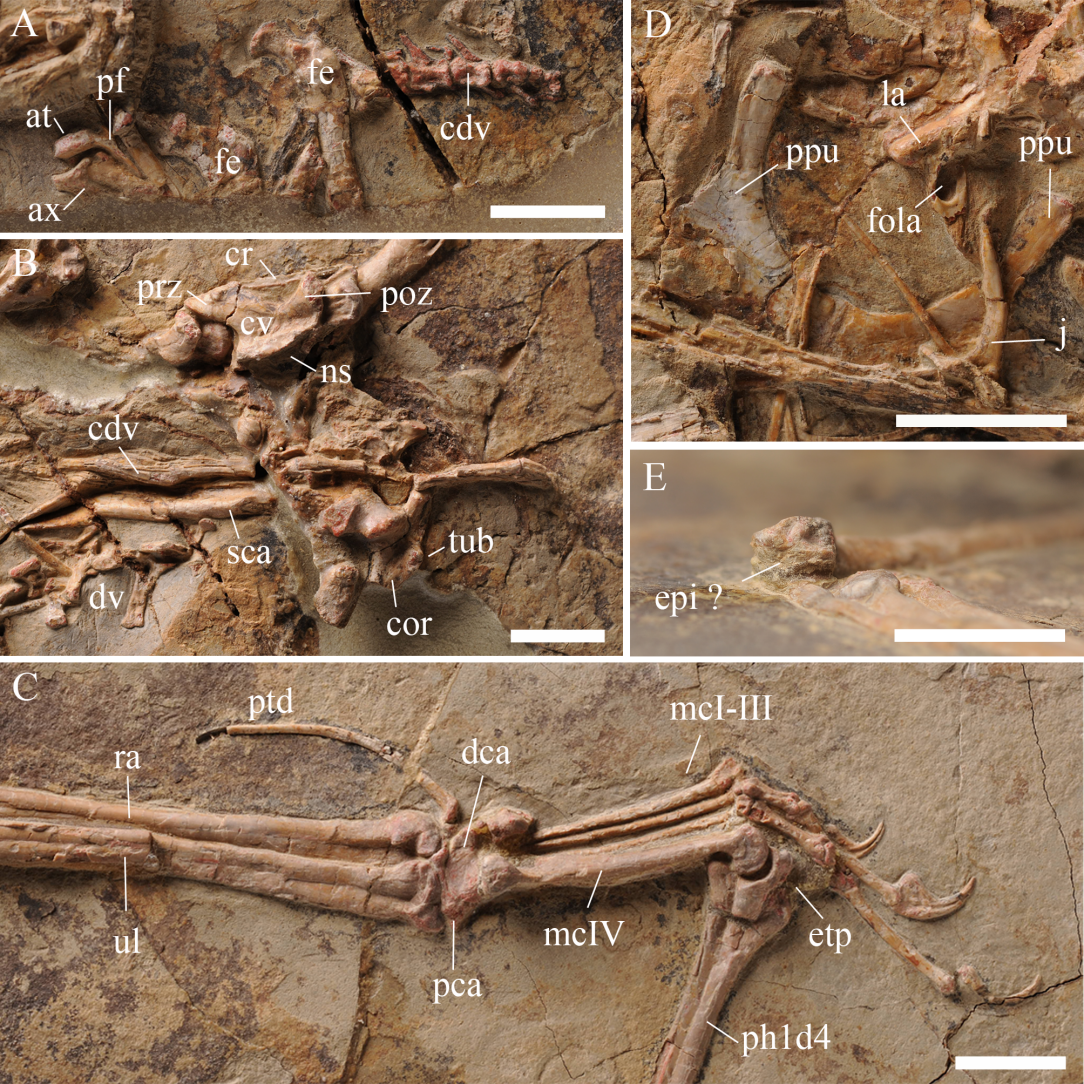
Details of the skeleton in IVPP V 17959. (A) Close up of the atlas, axis, anterior caudal vertebrae, and femur. (B) Close up of middle cervical vertebrae, dorsal vertebrae, posterior caudal vertebrae, scapula, and coracoid. (C) Close up of the left hand. (D) Close up of two fused prepubes. (E) Close up of the distal end of left humerus showing the unfused epiphysis. Scale bar: 10 mm. Abbreviation: at, atlas; ax, axis; cdv, caudal vertebra; cor, coracoid; cr, cervical rib; cv, cervical vertebra; dca, distal carpal series; dlca, distal lateral carpal; dv, dorsal vertebra; epi, epiphysis; etp, extensor tendon process; fe, femur; fola, foramen of lacrimal; la, lacrimal; mcI-IV, metacarpal 1–4; ns, neural spine; pca, proximal carpal series; pf, pneumatic foramen; ph1d4, first phalanx of digit 4; poz, postzygapophysis; ppu, prepubis; prz, prezygapophysis; ptd, pteroid; ra, radius; sca, scapula; tub, tubercle; ul, ulna; ?, uncertain.

An incomplete skeleton preserved in three-dimensions, housed in the Institute of Vertebrate Paleontology and Paleoanthropology (Chinese Academy of Sciences), Beijing, China, under the number IVPP V 17959 (Figs. 1–3).

***Locality and horizon***

Daxishan, Linglongta, Liaoning, China. Daohugou Bed or Tiaojishan Formation, Late Jurassic (Cheng, 2013; Wang et al., 2014a).

***Comments***

IVPP V 17959 is undoubtedly classified in the Wukongopteridae by sharing several cranial and postcranial features which are unique to the members of this clade among non-pterodactyloids (Wang et al., 2010) (see sections below).

IVPP V 17959 bears some features that differ from *Darwinopterus modularis*, *Darwinopterus linglongtaensis*, *Darwinopterus robustodens* and *Kunpengopterus sinensis*, including the presence of a bony crest limited to the anterior part of the premaxilla; an elongated nasoantorbital fenestra reaching about 60% of the length of the skull; a lacrimal bearing a large pear-shaped foramen, with the ventral margin wider than the dorsal margin; and the longest nasal process in the Wukongopteridae, with an angle of 90°between the proximal end of the process and the nasal.

The nasal foramen is absent in IVPP V 17959, which is different from *Darwinopterus linglongtaensis* and *Kunpengopterus sinensis* that preserve a foramen in the nasal. The lacrimal process of the jugal is thinner than the ones of *Darwinopterus modularis* and *Kunpengopterus sinensis*. IVPP V 17959 also differs from *Darwinopterus modularis* by having a wider ventral margin of the orbit and a greater distance between the posterior maxillary teeth.

However, the feet of IVPP V 17959 are incomplete and this is unfortunate because pedal morphology has been successfully used to distinguish wukongopterids (e.g., Wang et al., 2015). The only remarkable difference between IVPP V 17959 and *Wukongopterus lii*, which lacks a complete skull, is the adductor fossa that in IVPP V 17959 is about twice as long (relatively) as that of the former.

### Description and comparison

IVPP V 17959 is preserved in a slab of grey-white shale (Fig. 1). The specimen is composed of a partial skeleton with the skull and the lower jaw lacking the anterior tip, including several cervical, dorsal and caudal vertebrae, partial pectoral and pelvic girdle, and partial fore- and hind limbs. The skeleton is partially three-dimensionally preserved, differing from other pterosaur specimens discovered from the same locality which are much more compressed.

The skull is almost complete, lacking the rostral tip from the anterior margin of the nasoantorbital fenestra (Fig. 2). Part of the palate also is exposed; however, no details can be reported except that the choanae do not reach the middle part of the nasoantorbital fenestra.

The maxilla is poorly preserved. Only the anterior part can be recognized, which is fused with the premaxilla, forming the anterior margin of the nasoantorbital fenestra. The length of the nasoantorbital fenestra is estimated at about 60% of the skull length (Table 1), which maybe the longest in the Wukongopteridae (Wang et al., 2010; Lü et al., 2010; Lü et al., 2011a; Lü et al., 2011b; Wang et al., 2015).

**Table 1:** Measurements of IVPP V 17959 (in mm).

Bones	Length (mm)
Skull	145.0, est
Nasoantorbital fenestra	80.0, est
Lower jaw	130.0, est
Mandible symphysis	30.0, est
Humerus	41.0 r, est
Ulna	67.3 r
Metacarpal IV	27.6 l
	26.5 r
Wing phalange 1	55.3 r, est
Wing phalange 2	59.8 r, est
Metatarsal IV	15.1
Metatarsal V	2.7

**Notes.**

est estimate l left r right

The premaxilla of IVPP V 17959 has a crest that can be distinguished from *Kunpengopterus sinensis*, which preserves a flat dorsal margin of its skull (Wang et al., 2010). The crest is limited to the anterior part of premaxilla, not extending to the frontal as the crests do in *Darwinopterus modularis*, *Darwinopterus linglongtaensis*, and *Darwinopterus robustodens* (Lü et al., 2010; Lü et al., 2011b; Wang et al., 2010) (Fig. 2). From the preserved portion of the crest, it has a low and straight dorsal margin and backswept parallel ridges. The premaxilla bears a deep groove on the ventral side, which was also reported in *Darwinopterus robustodens* (Lü et al., 2011b).

The posterior part of premaxilla is missing and the right nasal is exposed in left view. The nasal has a fossa on the ventral side, but no foramen, which differs from *Darwinopterus linglongtaensis* and *Kunpengopterus sinensis* (Fig. 2; Wang et al., 2010). The proximal end of the process of the nasal forms a right-angle with the dorsal margin of the skull, which is different from the anteriorly declining nasal process in *Darwinopterus modularis*, *Darwinopterus linglongtaensis*, *Darwinopterus robustodens* and *Kunpengopterus sinensis* (Lü et al., 2010; Lü et al., 2011b; Wang et al., 2010). Although broken, it can be observed that both processes of the nasals fused together at the midline and formed one elongated process, which is longer than that of any other wukongopterids (Lü et al., 2010; Wang et al., 2010; Lü et al., 2011b).

The bones forming the skull roof are crushed and can barely be recognized. The right lacrimal was displaced ventrally and is not complete. The lacrimal is laterally inflated in the middle part, which bears a large pear-shaped opening (Fig. 3D) with the wider margin located ventrally. IVPP V 17959 is the only specimen reported to bear a lacrimal foramen in the Wukongopteridae. The ventral end of the lacrimal is pointed where it contacts the lacrimal process of the jugal.

In spite of being covered by other bones, the right jugal is complete and still in its original position. It bears four processes that contact with the maxilla, lacrimal, postorbital, and quadratojugal. The lacrimal process of jugal is thin, nearly vertical and pointed at its dorsal end. The lacrimal and maxilla processes of the jugal form the round posterior ventral margin of the nasoantorbital fenestra. The postorbital and quadratojugal processes of the jugal are partially covered by the right quadrate. Based on the uncovered part, the orbit has a boarder ventral margin compared with *Darwinopterus modularis* (Lü et al., 2010), with the posterior part being more rounded than the anterior part (Fig. 2).

The right quadrate is exposed on its medial side, is inclined posterodorsally, and forms an angle about 130°with the ventral margin of the skull. There are several lengthwise, slender fossae that can be observed in the middle side of the quadrate. The squamosal is badly broken and can only be recognized by its general shape and position (Fig. 2).

The lower jaw is well preserved, but lacks its rostral tip. The right lower jaw’s ramus is exposed on its medial side. The articular, angular, and surangular form a large adductor fossa, which is comparably twice as long as that of *Wukongopterus lii* (Wang et al., 2009). The articular bears a complicated cratered dorsal surface that connects with the quadrate (Fig. 2). From the preserved portion of the lower jaw, part of the mandible symphysis can be observed, showing that both dentaries are fused.

A few teeth are preserved in this specimen, and these teeth are much thinner than those preserved in other wukongopterids (e.g., Wang et al., 2009; Lü et al., 2010). Four teeth and alveoli are positioned ventral to the middle part of the nasoantorbital fenestra, the distance between which is about 5–6 times of the width of the alveolus.

The atlas and axis are completely separated (Fig. 3A). This is the first specimen of wukongopterids that exposes the atlas, which bears a slightly concave anterior and a convex posterior surface. The atlas’s neural arch inclines posterodorsally and thickens dorsally. The neural spine of the atlas is a laterally slender structure. The postzygapophysis of the atlas is smaller than that of the axis. A protrusion can be recognized at the same level as the postzygapophysis. A lateral foramen, which may be pneumatic based on the anatomy of other pterosaurs (e.g., Kellner & Tomida, 2000), is present between the arch and neural spine of the atlas. The axis is missing the posterior part of its centrum and has a concave anterior surface to connect with the atlas. The axis’s arch thickens dorsally like that of the atlas, but is laterally much thicker. Based on the preserved part of the neural spine of the axis, the spine is dorsally expanded more than that of the atlas. No foramen can be observed on the axis. Fragments of two middle cervical vertebrae are exposed near the left humerus (Fig. 3B). The middle cervical vertebra is elongated, procoelous, bearing slender cervical ribs and a low blade-like neural spine. The prezygapophysis is much bigger than the postzygapophysis. The centrum’s condyle extends posterior of the postzygapophysis.

The dorsal vertebrae are badly preserved, only several tall neural spines can be recognized. The caudal vertebrae are more complete than the cervical and dorsal ones, although they are divided into two regions and the end of the tail is preserved as an impression (Figs. 1, 3A and 3B). At least five free caudal vertebrae can be distinguished. They become gradually longer posteriorly (i.e., distally) and the first two are markedly shorter than any others (Fig. 3A). The next three free caudal vertebrae bear elongated postzygapophyses, which are much shorter than those of the posterior caudal vertebrae. Four elongated caudal vertebrae that are enclosed by rod-like bony extensions of the zygapophyses can be distinguished in the middle of the slab and some impressions are preserved (Fig. 1).

The pectoral girdles are missing except for partial right scapula and coracoid, which are fused together forming a scapulocoracoid (Figs. 1 and 3B). From the lateral side, it is evident that the scapula has a dorsoventrally compressed distal end. At both sides of the glenoid fossa, the scapula and coracoid bear a developed process. The glenoid fossa is comparably deep and concave. There is a well-developed lateral tubercle positioned close to the coracoid process.

In IVPP V 17959, the left wing is preserved from the humerus to a small portion of the proximal articulation of the third wing phalanx, whereas the right wing is preserved from the distal end of the radius and ulna to the first wing phalanx (Figs. 1, 2 and 3C). The proximal end and the deltopectoral crest of the left humerus is covered by the cervical vertebrae. The humeral shaft bends slightly ventrally. The right ulna and radius are straight and about the same width.

The left wrist bones are exposed in ventral view (Fig. 3C). Both the proximal and distal carpal series are fused, with the proximal syncarpal much shorter than the distal one. There is another small bone connected to the distal syncarpal that might be the distal lateral carpal. The left pteroid is a slender and curved bone. Although only preserved as an impression, the distal end of the pteroid is expanded, differing from the pointed one of *Darwinopterus linglongtaensis* (Wang et al., 2010).

The metacarpal regions from both sides of IVPP V 17959 are very complete (Fig. 1). Metacarpals I–III are much thinner than the wing metacarpal, a typical condition within Pterosauria, with the metacarpal I slightly thicker at its distal end than that of metacarpals II and III. The dorsal condyle of metacarpal IV shows a semicircular shape in lateral view (Fig. 3C). All metacarpal elements reach the carpus.

Manual digits I–III end in developed unguals. The extensor tendon process is fused with the wing finger on both sides (Fig. 3C). The first wing phalanx is bent slightly posteriorly (Fig. 1). Although the articular surfaces between the first and second wing phalanges are missing, from the preserved portions it is clear that the second wing phalanx is longer than the first (Table 1).

Two prepubes are the only preserved portions of the pelvic girdle, which was displaced to lie near the nasoantorbital fenestra (Fig. 2). They bear a comparably broad shaft and are expanded anteriodistally. The posterior margins form a smooth arc in dorsoventral view. They are fused with only a small portion of their suture visible at the posterior end (Fig. 3D).

Only the proximal ends of both femora are preserved (Fig. 3A). The femoral head has a longer neck than that of *Kunpengopterus sinensis* (Wang et al., 2010). Metatarsal IV and V are preserved, as well as fragments of the proximal and distal tarsals. Metatarsal IV is about six times longer than metatarsal V (Table 1).

## Discussion

**Figure 4:**
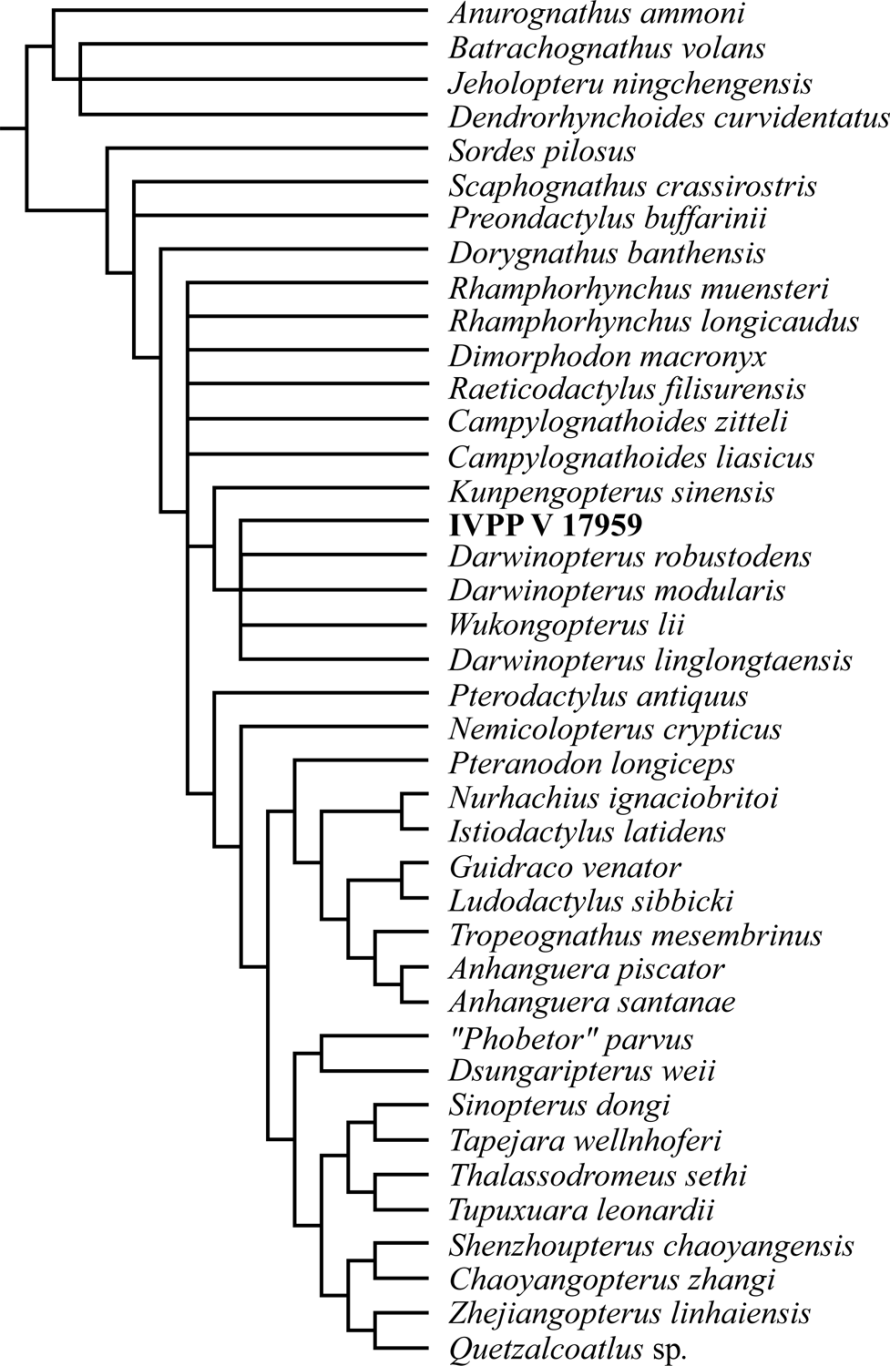
Phylogenetic relationships of IVPP V 17959. The new specimen is indicated with bold characters. Tree length, 260; CI, 0.593; RI, 0.832.

In order to assess the phylogenetic position of IVPP V 17959, we performed a phylogenetic analysis, mainly based on Wang et al. (2012), with some changes made using previous studies. We obtained five most parsimonious cladograms and the strict consensus tree is shown in Fig. 4 (see Supplemental Information 1 for details). The basal portion of the cladogram (i.e., non-Pterodactyloidea) is highly unresolved. The Wukongopteridae, however, are supported as monophyletic, with *Kunpengopterus* occupying a basal position, followed by an unresolved polytomy that includes all remaining members of this clade and the new specimen. Characters supporting the monophyly of the Wukongopteridae that were also observed in IVPP V 17959 are as follows: presence of a confluent nasoantorbital fenestra (Character 7.1); presence of a free nasal process (Character 24.1); lacrimal process of the jugal subvertical (Character 31.1); mid-cervical vertebrae are elongated (68.1); neural spines of the mid-cervical vertebrae are low (Character 70.1).

IVPP V 17959 lacks one unique character of *Kunpengopterus*, which is the presence of a pneumatic on the nasal process (Character 28.1).

IVPP V 17959 shares the following features with the remaining wukongopterids: a confluent nasoantorbital fenestra longer than 40% of the skull length (Character 8.1), presence of a premaxillary crest (Character 16.1), and a thin lacrimal process of the jugal (Character 30.1).

Although the exact relationship between IVPP V 17959 and other wukongopterids is still unknown, the new specimen differs from *Wukongopterus lii*, *Darwinopterus modularis*, *Darwinopterus linglongtaensis*, and *Darwinopterus robustodens* by the following features: a premaxillary sagittal crest confined to the anterior portion of the skull (Character 17.0) and the presence of a long free nasal process (Character 26.0). Despite the fact that anatomically there are arguments to regard this specimen as representing a new species, we refrain from naming IVPP V 17959 due to the incompleteness of the skull and the feet, both of which are regions that have provided important characters for taxonomic distinctions within the Wukongopteridae (e.g., Wang et al., 2010). Furthermore, the phylogenetic analysis performed here did not conclusively set this specimen apart from previously named taxa and therefore we prefer to wait for new discoveries that might elucidate the taxonomic identity of this material.

Nonetheless regarding phylogeny, the fusion of the atlas and axis has been used as a pterodactyloid character (e.g., Bennett, 1994; Kellner, 2003; Wang et al., 2012; Andres & Myers, 2013). In many pterodactyloid pterosaurs, the atlas and axis are fused in adults, including *Pteranodon*, *Nyctosaurus*, *Dsungaripterus*, *Azhdarcho* and *Quetzalcoatlus* (Bennett, 2001). IVPP V 17959 represents an adult individual, with the atlas and axis clearly separated, which indicates that these bones might not fuse at all. This is another example of a character state in Wukongopteridae that seems to separate it from pterodactyloid pterosaurs.

In accordance with previous research on the ontogeny of Pterosauria (Bennett, 1995; Kellner & Tomida, 2000; Kellner et al., 2013; Kellner, 2015), IVPP V 17959 shows evidence that it was a skeletally mature animal when it perished, since several bones are fused. Recently, Kellner (2015) introduced six ontogenetic stages (OS1–OS6) based on the fusion of different elements to separate pterosaurs regarding their ontogenetic development. Based on this classification, IVPP V 17959 had reached the OS5 state (i.e., maturity) because shows an extensor tendon process fused with the first wing finger’s phalanx. Observing the distal end of the humerus, a small projected element is here interpreted as the epiphysis which was still unfused (Fig. 3E), suggesting that IVPP V 17959 had not yet reached OS6 (see Kellner, 2015). Still regarding ontogeny, the prepubes show that they were fused (Fig. 3D) at least at this stage (OS5), if not earlier.

It is interesting to point out that the holotype of *Darwinopterus robustodens* (41HIII-0309A) also represents an individual with a similar ontogenetic stage, what is based on the fused condition of the pelvic elements where the ilium is fused with the puboischiadic plate (Lü et al., 2011b; Kellner, 2015). However, in 41HIII-0309A the premaxilla crest extends over the skull roof (Lü et al., 2011b) whereas in IVPP V 17959 it ends at the anterior half of the nasoantorbital fenestra. Based on the length of the ulna, IVPP V 17959 is about 17% smaller than 41HIII-0309A. Even so, the variation of the crest size and location between those two specimens does not appear to be ontogenetic, and we do not expect that smaller individuals of *Darwinopterus robustodens* might show a crest similar to IVPP V 17959. The fact that the holotype of another crested wukongopterid, *Darwinopterus linglongtaensis* (IVPP V 16049) is about 14% smaller than IVPP V 17959 (also based on the length of the ulna), but has a crest extending to the skull roof, corroborates this interpretation.

Lü et al. (2011a) described a crest-less wukongopterid specimen (ZMNH M8802) associated with an egg and assigned it to *Darwinopterus*. Furthermore, they argued that this was evidence for sexual dimorphism, using other specimens with crests that they regarded as representing males, implying that all belonged to the species *D. modularis*. Subsequently, Lü et al. (2011b) came to the conclusion that one of the males actually belonged to a distinct species. However, Wang et al. (2010) described a crestless wukongopterid (*Kunpengopterus sinensis*) that, besides the crest, presented several other anatomical differences from the crested forms. These include the shape of the posterior region of the skull, the lacrimal process of the jugal and features found in the foot. Furthermore, recent studies of pterosaur bone beds that are expected to include both males and females have conclusively shown that at least in one species, premaxillary crests are present in posthatchlings (Manzig et al., 2014). Moreover, in an amazing site where dozens of skeletons were found associated with eggs (indication of the presence of sexually mature females), individuals with variations of crests were identified showing that not the presence of the crest but its expression might be sexually dimorphic (Wang et al., 2014b). The discovery of the present wukongopterid specimen (IVPP V 17959) where the crest is present and varies in shape from others supports this interpretation.

## Conclusion

The discovery of a new wukongopterid specimen (IVPP V 17959) sheds new light on the anatomy of wukongopterids, including ontogenetic assessment. IVPP V 17959 can be classified in the ontogenetic stage 5. It further shows that fusion of the prepubes has occurred at least at this stage, if not earlier. The unfused atlas and axis at this ontogenetic stage also suggests that these bones never fused in the Wukongopteridae, a common feature within other non-pterodactyloids.

The new specimen shows a premaxillary crest that differs from other wukongopterids of comparable ontogenetic stages. This feature, along with other studies, corroborates with the hypothesis that the presence or absence of cranial crests are diagnostic and not a result of sexual dimorphism. More work on basal pterosaurs is needed to provide a more robust hypothesis of their relationships, but wukongopterids remain supported as a valid clade.

##  Supplemental Information

10.7717/peerj.2177/supp-1 Supplemental Information 1 Phylogeny analysis data of IVPP V 17959 Character list and data matrix.

## Additional Information and Declarations

### Data Availability

The following information was supplied regarding data availability: The research in this article did not generate any raw data.
