# Supplementary material for: New information on the Wukongopteridae (Pterosauria) revealed by a new specimen from the Jurassic of China
Source: PeerJ. 2016 Jul 7;4:e2177. doi: 10.7717/peerj.2177 (PMC4941781; doi:10.7717/peerj.2177)
Supplement: Supplemental Information 1 — Character list and data matrix. [file peerj-04-2177-s001.docx]

**Supplemental Information**

CHARACTER LIST (per anatomical region)

SKULL

1. Dorsal margin of the skull: 0 - straight or curved downward 1 - concave 2 - only rostrum curved upward

2. Upper and lower jaw: 0 - laterally compressed 1 - comparatively broad

3. Rostral part of the skull anterior to the external nares: 0 - reduced 1 - elongated (less than half of skull length) 2 - extremely elongated (more than half of skull length)

4. Rostral end of premaxillae/maxillae downturned: 0 - absent 1 - present

5. Position of the external naris: 0 - above the premaxillary tooth row 1 - displaced posterior to the premaxillary tooth row

6. Process separating the external nares: 0 - broad 1 - narrow

7. External naris and antorbital fenestra: 0 - separated 1 - confluent forming a nasoantorbital fenestra

8. Naris and antorbital fenestra: 0 - shorter than 40% of the skull length 1 - longer than 40% of the skull length

9. Posterior margin of antorbital (or nasoantorbital) fenestra: 0 - straight 1 - concave

10. Nasoantorbital (or antorbital) fenestra extending dorsal to the orbit 0 - absent 1 - present

11. Shape of the orbit:

0 - subcircular 1 - circular 2 - circular, with open ventral margin 3 - piriform (elongated)

12. Orbit comparatively small and positioned very high in the skull: 0 - absent 1 - present

13. Position of the orbit relative to the nasoantorbital fenestra (naris + antorbital fenestra): 0 - same level or higher 1 - orbit lower than the dorsal rim of the nasoantorbital fenestra

14. Suborbital opening: 0 - absent 1 - present

15. Lower temporal fenestra: 0 - piriform, with ventral portion wider than dorsal 1 - piriform, with dorsal portion wider than ventral 2 - reduced (slit-like)

16. Premaxillary sagittal crest: 0 - absent 1 - present

17. Premaxillary sagittal crest, position: 0 - confined to the anterior portion of the skull 1 - starting anterior to the anterior margin of the nasoantorbital fenestra, extending beyond occipital region 2 - starting at about the anterior margin of the nasoantorbital fenestra, reaching the skull roof above the orbit but not extending over the occipital region 3 - starting close or at the anterior portion of the skull and extended over the occipital region 4 - starting at the posterior half of the nasoantorbital fenestra. 5 - starting at the middle portion of the nasoantorbital fenestra, extending above the occipital region.

18. Premaxillary sagittal crest shape: 0 - striated, low with a nearly straight dorsal margin 1 - striated, high, spike-like

2 - round dorsal margin, blade-shaped3 - smooth, expanded anteriorly and forming a low rod-like extension posteriorly4 - smooth, starting low anteriorly and very expanded posteriorly

19. Expansion on the anterior part of the premaxillary sagittal crest: 0 - absent 1 - present

20. Elongated dorsal premaxillary spike-like extension 0 - absent 1 - present

21. Expansion of the premaxillary tip: 0 - absent 1 - present, with premaxillary end high 2 - present, with premaxillary end dorsoventrally flattened.

22. Posterior ventral expansion of the maxilla: 0 - absent 1 - present

23. Maxilla-nasal contact 0 - broad 1 - absent

24. Free nasal process: 0 - absent 1 - present

25. Free nasal process position:

0 - placed laterally1 - placed medially

26. Free nasal process size:

0 - long, almost reaching the ventral margin of the skull1 - short 2 - knob-like (extremely reduced)

27. Free nasal process orientation:

0 - directed anteroventrally1 - directed ventrally

28. Foramen on nasal process: 0 - absent 1 - present

29. Lacrimal extensively fenestrated 0 - absent 1 - present

30. Lacrimal process of the jugal thickness: 0 - broad 1 - thin

31. Lacrimal process of the jugal inclination:

0 - inclined anteriorly1 - subvertical2 - inclined posteriorly

32. Pronounced ridge on the lateral side of the jugal 0 - absent 1 - present

33. Anterior portion of the frontal rugose: 0 - absent

1 - present

34. Bony frontal crest: 0 - absent 1 - present

35. Bony frontal crest, position: 0 - confined to the posterior end of the skull 1 - starting above the orbit 2 - starting on the posterior half of the nasoantorbital fenestra

36. Bony frontal crest, shape:

0 - reduced and blunt 1 - short and spike-like, dorsally deflected 2 - narrow, directed posteriorly 3 - very high and broad, at least doubling the height of the skull above the orbit, directed posteriorly 4 - high, broad base and fan-shaped

5 - casque-like

37. Bony parietal crest: 0 - absent 1 - present

38. Bony parietal crest shape: 0 - blunt 1 - constituting the base of the posterior portion of the cranial crest.

39. Posterior region of the skull rounded with the squamosal displaced ventrally: 0 - absent 1 - present

40. Position of the quadrate relative to the ventral margin of the skull: 0 - vertical or subvertical 1 - inclined about 120° backwards 2 - inclined about 150° backwards

41. Position of the articulation between skull and mandible: 0 - under the posterior half of the orbit or further backwards 1 - under the middle part of the orbit 2 - under the anterior half of the orbit or further anterior

42. Helical jaw joint: 0 - absent 1 - present

43. Supraoccipital: 0 - does not extend backwards 1 - extends backwards

44. Foramen pneumaticum piercing the supraoccipital: 0 - absent 1 - present

45. Expanded distal ends of the paroccipital processes: 0 - absent 1 - present

46. Palatal ridge: 0 - absent 1 - discrete, tapering anteriorly 2 - strong, tapering anteriorly 3 - strong, confined to the posterior portion of the palate

47. Slight expansion of the palate close to the anterior opening of the nasoantorbital (or naris + antorbital) fenestra: 0 - absent 1 - present

48. Maxilla excluded from the internal naris: 0 - absent 1 - present

49. Opening between pterygoids and basisphenoid (interpterygoid opening): 0 - absent or very reduced 1 - present and larger than subtemporal fenestra 2 - present but smaller than subtemporal fenestra

50. Basisphenoid: 0 - short 1 - elongated

51. Mandibular symphysis: 0 - absent or very short 1 - present, at least 30% of mandible length

52. Step-like dorsal margin of the dentary in lateral view: 0 - absent 1 - present

53. Anterior tip of the dentary downturned: 0 - absent 1 - present

54. Dentary bony sagittal crest: 0 - absent 1 - present

55. Dentary bony sagittal crest, position: 0 - confined to the anterior third of the lower jaw 1 - extending close to the middle partion of the jaw

56. Dentary bony sagittal crest shape: 0 - small projection

1 - blade-like2 - elongated ridge-like3 - deep, broad in lateral view4 - shallow

57. Position and presence of teeth: 0 - teeth present, evenly distributed along the jaws 1 - teeth absent from the anterior portion of the jaws 2 - teeth confined to the anterior part of the jaws 3 - jaws toothless

58. Largest maxillary teeth positioned posteriorly: 0 - absent 1 - present

59. Variation in the size of the anterior teeth with the 5th and 6th smaller than the 4th and 7th: 0 - absent 1 - present

60. Teeth with a broad and oval base: 0 - absent 1 - present

61. Teeth finely serrated:

0 - present 1 - absent

62. Peg-like teeth: 0 - absent 1 - present, 15 less on each side of the jaws 2 - present, more than 15 on each side of the jaws

63. Laterally compressed and triangular teeth:

0 - absent

1 - presentAXIAL SKELETON

64. Atlas and axis: 0 - unfused 1 - fused

65. Notarium: 0 - absent 1 - present

66. Postexapophyses on cervical vertebrae: 0 - absent 1 - present

67. Lateral pneumatic foramen on the centrum of the cervical vertebrae: 0 - absent 1 - present

68. Midcervical vertebrae: 0 - short, sub-equal in length 1 - elongated 2 - extremely elongated

69. Cervical ribs on midcervical vertebrae: 0 - present 1 - absent

70. Neural spines of the mid-cervical vertebrae, height: 0 - tall 1 - low 2 - extremely reduced or absent

71. Neural spines of the mid-cervical vertebrae, shape: 0 - blade-like 1 - spike-like 2 - ridge

72. Number of caudal vertebrae: 0 - more than 15 1 - 15 or less

73. Caudal vertebrae with elongated zygapophyses forming rod-like bony processes: 0 - absent 1 - present

74. Proximal caudal vertebrae with duplex centra: 0 - absent 1 - present

PECTORAL GIRDLE

75. Length of the scapula: 0 - subequal or longer than coracoid 1 - scapula shorter than coracoid (1 > sca/cor > 0.80) 2 - substantially shorter than coracoid (sca/cor < 0.80)

76. Proximal surface of scapula: 0 - elongated 1 - sub-oval

77. Shape of scapula: 0 - elongated 1 - stout, with constructed shaft

78. Coracoidal contact surface with sternum: 0 - flattened 1 - oval

79. Coracoidal contact surface with sternum: 0 - no developed articulation surface 1 - articulation straight or slightly concave 2 - articulation strongly concave

80. Posterior expansion on articulation surface of the coracoid with the sternum: 0 - absent 1 - present

81. Deep coracoidal flange: 0 - absent 1 - present

82. Broad tubercle on ventroposterior margin of coracoid: 0 - absent 1 - present

83. Cristospine:

0 - absent

1 - shallow and elongated 2 - deep and shortFORELIMB

84. Proportional length of the humerus relative to the metacarpal IV (hu/mcIV): 0 - hu/mcIV > 2.50 1 - 1.50 < hu/mcIV < 2.50 2 - 0.40 < hu/mcIV < 1.50 3 - hu/mcIV < 0.40

85. Proportional length of the humerus relative to the femur (hu/fe): 0 - hu/fe < 0.80 1 - 1.4 > hu/fe > 0.80 2 - hu/fe > 1.40

86. Proportional length of the humerus plus ulna relative to the femur plus tibia (hu+ul/fe+ti): 0 - humerus plus ulna about 0.80% or less of femur plus tibia length (hu+ul/fe+ti < 0.80) 1 - humerus plus ulna larger than 0.80% of femur plus tibia length (hu+ul/fe+ti > 0.80)

87. Pneumatic foramen on the ventral side of the proximal part of the humerus: 0 - absent 1 - present

88. Pneumatic foramen present on dorsal side of the proximal part of the humerus: 0 - absent 1 - present

89. Deltopectoral crest of the humerus: 0 - reduced, positioned close to the humerus shaft 1 - enlarged, proximally placed, with almost straight proximal margin 2 - enlarged, hatchet shaped, proximally placed 3 - enlarged, hatched shaped, positioned further down the humerus shaft 4 - enlarged, warped 5 - long, proximally placed, curving ventrally

90. Medial (= ulnar) crest of the humerus: 0 - absent or reduced 1 - present, directed posteriorly 2 - present, massive, with a developed proximal ridge

91. Distal end of the humerus: 0 - oval or D-shaped 1 - subtriangular

92. Proportional length of the ulna relative to the metacarpal IV (ul/mcIV): 0 - ulna 3.6 times longer than metacarpal IV (ul/mcIV > 3.6) 1 - length of ulna between 3.6 and two times the length of metacarpal IV (3.6 > ul/mcIV > 2)

2 - ulna between two times and the same length of metacarpal IV (2 > ul/mcIV > 1)

3 - ulna about the same length or smaller than metacarpal IV (ul/mcIV < 1)

93. Diameter of radius and ulna: 0 - subequal 1 - diameter of the radius about half that of the ulna 2 - diameter of the radius less than half that of the ulna

94. Distal syncarpals, shape: 0 - irregular 1 - from a rectangular unit 2 - form a triangular unit

95. Pteroid: 0 - absent 1 - shorter than half the length of the ulna 2 - longer that half the length of the ulna

96. Metacarpals I - III: 0 - articulating with carpus 1 - metacarpal I articulates with carpus, metacarpals II and III reduced 2 - not articulating with carpus

97. Proportional length of the first phalanx of manual digit IV relative to the metacarpal IV (ph1d4/mcIV): 0 - both small and reduced 1 - both enlarged with ph1d4 over four times the length of mcIV (ph1d4/mcIV>4.0)

2 - both enlarged with ph1d4 over two times, but less than four times the length of mcIV (2.0<ph1d4/mcIV<4.0) 3 - both enlarged with ph1d4 about or less than two times the length of mcIV (ph1d4/mcIV<2.0) about 2 or smaller.

98. Proportional length of the first phalanx of manual digit IV relative to the tibiotarsus (ph1d4/ti): 0 - ph1d4 reduced 1 - ph1d4 elongated and less than twice the length of ti (ph1d4/ti smaller than 2.00) 2 - ph1d4 elongated about or longer than twice the length of ti (ph1d4/ti subequal/larger than 2.00)

99. Proportional length of the second phalanx of manual digit IV relative to the first phalanx of manual digit IV (ph2d4/ph1d4): 0 - both short or absent 1 - elongated with second phalanx about the same size or longer than first (ph2d4/ph1d4 larger than 1.00) 2 - elongated with second phalanx up to 30% shorter than first (ph2d4/ph1d4 between 0.70 - 1.00) 3 - elongated with second phalanx more than 30% shorter than first (ph2d4/ph1d4 smaller than 0.70)

100. Proportional length of the third phalanx of manual digit IV relative to the first phalanx of manual digit IV (ph3d4/ph1d4): 0 - both short or absent 1 - ph3d4 about the same length or larger than ph1d4 2 - ph3d4 shorter than ph1d4

101. Proportional length of the third phalanx of manual digit IV relative to the second phalanx of manual digit IV (ph3d4/ph2d4): 0 - both short or absent 1 - ph3d4 about the same size or longer than ph2d4 2 - ph3d4 shorter than ph2d4

102. Proportional length of the forth phalanx of manual digit IV relative to the first phalanx of manual digit IV (ph4d4/ph1d4): 0 - both short or absent 1 - both elongated, with the forth phalanx the longer than the first (ph4/d4/ph1d4>1.00)

2 - both elongated with the forth phalanx the same length or shorter, but longer than 35% the length of the first (1.00>ph4d4/ph1d4>0.35)

3 - both elongated with the forth phalanx less than 35% the length of the first (ph4d4/ph1d4<0.35)HINDLIMB

103. Proportional length of the femur relative to the metacarpal IV (fe/mcIV): 0 - femur about twice or longer than metacarpal IV(fe/mcIV > 2.00) 1 - femur longer but less than twice the length of metacarpal IV (1.00 < fe/mcIV < 2.00) 2 - femur about the same length or shorter than metacarpal IV (fe/mcIV < 1.00)

104. Length of metatarsal III: 0 - more than 30% of tibia length 1 - less than 30% of tibia length

105. Fifth pedal digit: 0 - with four phalanges 1 - with 2 phalanges 2 - with 1 or no phalanx (extremely reduced)

106. Last phalanx of pedal digit V: 0 - reduced or absent 1 - elongated, straight 2 - elongated, curved 3 - elongated, very curved (boomerang shape)

DATA MATRIX

***Ornithosuchus longidens***0000000-00 000000---0 0000----00 0000--0-00 0000000000 0000--0000 0000000000 0000000000 0000000000 0000000000 000000***Herrerasaurus ischigualastensis***0000000-00 000000---0 0000----00 0000--0-00 0000000000 0000--0000 0000000000 0000000000 0000000000 0000000000 000020

***Scleromochlus taylori*** 000?0?0-?0 ?000?0---0 0000----?? ???0--??00 0?????0??? ?0?0--00?0 ?0??0??00? ?000????00 ????00??0? ??0?0????? ?0????

***Anurognathus ammoni*** 010001???0 ?????0---0 00?0----0? ?0?0--0-?? ??????0??? 0000--0000 110?0??0?? ?100??0??? ???011??10 000?1011?? ??001?

***Batrachognathus volans***

?10001???? ?????0---0 00???????? ??0--????? ??????00?? 0?00--0000 110??0?0?0 0????00010 ????21??10 ?????????? ??????

***Dendrorhynchoides curvidentatus***

010001???? ?????0---0 00???????? ???0--???? ??????0??? ??00--0000 110?0??00? ?1000?0010 00?021??1? ?00?101122 2?00??

***Jeholopteru ningchengensis***

010001???? ?????0---0 00???????? ???0--0-?? 0?????0??? ??00--0000 110?0??01? ?10?000010 00?021??1? ?00?101122 231011

***Sordes pilosus***

0010100-00 0000?0---0 0000----?0 0000--0-01 0?0?0?0??? 0000--0000 1?0?00?000 0010000010 00?011??10 000?102111 120013

***Preondactylusbuffarinii***

0010100-???000?0---00000----?????0--0-0?0?????0???0000--0?00?00?0??0???0???????????111??1??10?1021111200??

***Scaphognathus crassirostris***

0010100-00000000---00000----000000--0-01000?000010?000--0000100?00000000100000100011110010010?102111110013

***Dorygnathusbanthensis***

0010100-00000000---00000----000000--0-01000?0?0?1?1000--0000100?0??00000100000100??1110020010?102111121013

***Dimorphodonmacronyx***

0010100-00300010---00000----000000--0-000?0?0?0???0000--0000100?00000000100000100??1110010011?1021111?1011

***Raeticodactylus filisurensis***

0010100-0000001111000000----0000??????010?????0???0001030000100?0??00???????????????21??20???????1211??0??

***Campylognathoidesliasicus***

0010100-00000010---00000----000000--0-01000?0001100010--0000100000000000100000100011110020011?101212221010

***Campylognathoideszitteli***

0010100-00000010---00000----000000--0-010?0???0???0010--00001000000000001000001000?1???12??11?101212220010

***Rhamphorhynchuslongicaudus***

0010100-00000010---00000----000000--0-01100?000??01000--0000100000?00000100000100011110020011?102222221012

***Rhamphorhynchus muensteri***0010100-00 000010---0 0000----00 0000--0-01 1000000110 1010--0000 1000000000 0010000010 0011110020 011?101222 221012

***Pterodactylus antiquus***0010101000 000010---0 0011001000 1000--0-12 1?0?000??? 1000--0000 1200000111 0100000010 0012110050 021?103122 222020

***Nemicolopterus crypticus*** 0010101000 0000?0---0 0011110001 1000--0-0? 2?????0??? 1000--3000 100?0?011? ?100000??? 0???11?15? ?2????31?2 2?2120

***Pteranodon longiceps***1020101010 300010---0 001112-000 1101221101 2101000121 1000--3000 1001111110 1101110120 0012111041 1312223222 232020

***Istiodactylus latidens***0010101100 2???10---0 0?11110?01 21?0--0-01 2?01?00??1 0000--2000 101?111??0 1???111020 002?1?0141 1?22?????? ??????

***Nurhachius ignaciobritoi***0010101100 ?000?0---0 001?????01 110??????1 2?????0??? ?000--2000 10101??110 1???1?1020 0?22110?41 ?222?1312? ??21??

***Tropeognathus mesembrinus***0010101000 3000110200 101?????00 1101001001 2101020121 1001010000 100??????? ?????????? ?????????? ?????????? ??????

***Anhanguera santanae***0010101010 3000110200 1011100100 1101001001 2101010121 100??10010 1001011110 11??211021 00????0141 1?22?1???? ??????

***Anhanguera piscator***0010101010 3000110200 1011100100 1101001001 21010?0121 1001010010 1001011110 1101211121 0022110141 1222?1???? ??2120

***Ludodactylus sibbicki***0010101000 000010---0 ?011100100 01011?1101 110?0?0??? 1000--0010 100??????? ?????????? ?????????? ?????????? ??????

***Guidraco venator* gen et sp nov.**

0020101010 300010---0 ?01?????00 0101151101 110?0?0??? 1000--0000 100??1101? ?????????? ?????????? ?????????? ??????

***Dsungaripterus weii***2010101000 1101111100 0110----00 1001211101 2111110121 1000--1101 1001111110 010?000??? 0?2210??5? 0311?13122 2?2120

***"Phobetor" parvus***

0010101000 1101111100 0110----00 1001211101 2?1?1?0??? 1000--1101 100??????0 ?????????? ?????????? ???1?????? ??????

***Quetzalcoatlus* sp*.***0010101000 3010?14?00 0010----00 100??????1 21???001?? 1000--3000 1001110212 2?0?000020 10?20?1052 0311??3132 2?2?20

***Zhejiangopterus linhaiensis***0010101000 3010?0---0 0010----00 1000--0-01 2?1???0??? ?000--3000 10011??212 2?0?0002?? 10?200??5? ?31?2?313? ??2???

***Chaoyangopterus zhangi*** 1010101??? ?0???????0 00???????? ?????????? ??????0??? 1000--3000 100?010110 0???000??? 00?200???? ?31?2?3122 2321?0

***Shenzhoupterus chaoyangensis***1010101101 301020---0 001?????0? 2001241101 2?1???0??? 1000--3000 100?01011? ????0??0?? 0??200???? ?31???3122 2321??

***Tupuxuara leonardii***0010101100 3010213400 001?????01 1001231101 2111130121 1001123000 1001111?10 0???000020 0122101052 0311?3213? ??2???

***Thalassodromeus sethi***0010101100 3010213400 001112-001 1001231101 2111130121 1000--3000 100??????? ?????????? ?????????? ?????????? ??????

***Tapejara wellnhoferi***0011101100 3010213300 0011111011 1001221101 2011101121 1101133000 100?011?10 0?0?000020 01?2101152 03112?31?? ??2120

***Sinopterus dongi*** 0011101100 3010?13300 0011100011 1011211101 2?1???1??? ?101143000 100?010110 0???000020 0??2111?52 ?31?2?3122 2321??

***Darwinopteruslinglongtaensis***

0010101100000011200000110101011?00--0-11100?0?????0000--00001?0?0??101001?00001000?111?120010?102111111013

***Wukongopteruslii***

001010??????????????00????????????????????????????0000--0000120?0?0101001?000???0??111?0???10?102111111013

***Darwinopterusmodularis***

0010101100000011200000110100011000--0-11100?0?????0000--0000120?0??1?1001?000???0??1????2?010???2????1??13

***Darwinopterusrobustus***

0010101100000011200000110100011000--0-11100?0?????0000--00001?0?0??101001?00001000?111?0?0010?102111111013

***Kunpengopterussinensis***

00101010000000?0---000110101001?00--0-11100?0?????0000--0??01?0?000101001?00001000?111???0010?102111121012

**IVPP V 17959**

00?01011000000?100?0?01100?0011?00--0-1110????????00????0??01?00?0?101001??00??????1?????0010?102?1???????
